# Thymoquinone Attenuates Cardiomyopathy in Streptozotocin-Treated Diabetic Rats

**DOI:** 10.1155/2018/7845681

**Published:** 2018-10-30

**Authors:** Mustafa S. Atta, Ali H. El-Far, Foad A. Farrag, Mohamed M. Abdel-Daim, Soad K. Al Jaouni, Shaker A. Mousa

**Affiliations:** ^1^Department of Physiology, Faculty of Veterinary Medicine, Kafrelsheikh University, Kafrelsheikh 33516, Egypt; ^2^Department of Biochemistry, Faculty of Veterinary Medicine, Damanhour University, Damanhour 22511, Egypt; ^3^Department of Anatomy and Embryology, Faculty of Veterinary Medicine, Kafrelsheikh University, Kafrelsheikh 33516, Egypt; ^4^Department of Pharmacology, Faculty of Veterinary Medicine, Suez Canal University, Ismailia 41522, Egypt; ^5^Hematology/Pediatric Oncology, King Abdulaziz University Hospital and Scientific Chair of Yousef Abdullatif Jameel of Prophetic Medicine Application, Faculty of Medicine, King Abdulaziz University, Jaddah 21589, Saudi Arabia; ^6^Pharmaceutical Research Institute, Albany College of Pharmacy and Health Sciences, Rensselaer, NY 12144, USA

## Abstract

Diabetic cardiomyopathy is a diabetic complication due to oxidative stress injuries. This study examined the protecting influence of thymoquinone (TQ) on diabetes-caused cardiac complications. The intracellular means by which TQ works against diabetes-caused cardiac myopathy in rats is not completely understood. In this study, Wistar male rats (*n* = 60) were assigned into four groups: control, diabetic (diabetes induced by IP infusion of streptozotocin, 65 mg/kg), diabetic + TQ (diabetic rats given TQ (50 mg/kg) administered once per day by stomach gavage), and TQ (50 mg/kg) for 12 weeks. TQ supplementation appreciably recovered the cardiac parameters alongside significant declines in plasma nitric oxide concentrations and total superoxide dismutase (T.SOD) activities. Importantly, TQ downgraded expression of cardiac-inducible nitric oxide synthase in addition to significantly upregulating vascular endothelial growth factor and erythropoietin genes and nuclear factor-erythroid-2-related factor 2 (Nrf2) protein. TQ normalized plasma triacylglycerol and low-density lipoprotein-cholesterol and significantly improved the high-density lipoprotein-cholesterol levels. Additionally, TQ administration improved the antioxidant ability of cardiac tissue via significantly increased cardiac T.SOD and decreased cardiac malondialdehyde levels. Oral supplementation with TQ prevented diabetic-induced cardiomyopathy via its inhibitory effect on the E-selectin level, C-reactive protein, and interleukin-6. The TQ protecting effect on the heart tissue was shown by normalization of the plasma cardiac markers troponin I and creatine kinase. This experiment shows the aptitude of TQ to protect cardiac muscles against diabetic oxidative stress, mainly through upregulation of Nrf2, which defeated oxidative damage by improvement of the antioxidant power of cardiac muscle that consequently protected the cardiac muscles and alleviated the inflammatory process.

## 1. Introduction

Diabetes mellitus (DM) is a metabolic ailment that occurs due to different factors including either genetic or environmental influences. DM is distinguished by disturbances in insulin metabolism that consequently alter carbohydrates, lipids, and protein metabolisms [1]. A cascade of myocardial variations that occur in DM with fibrosis, hypertrophy, and microcirculatory imperfections characterizes diabetic cardiomyopathy. These circulatory complaints hinder the heart efficiency, then concomitantly result in cardiac failure [2]. Diabetic-induced cardiac complication is distinguished by myocardial functional alterations in which oxidative stresses are the main cause [3]. DM-generated reactive oxygen species (ROS) that led to injuries to cellular structures subsequently led to functional, structural, and metabolic impairments [4]. ROS creation triggers cardiomyocytes' necrosis besides apoptosis, which induces cardiac alterations and dysfunction. Therefore, the cellular antioxidant molecules try to abolish the harmful effect of ROS to maintain cellular integrity [5]. Oxidative stress-associated pathological processes can be attenuated by the nuclear factor-erythroid-2- (NF-E2-) related factor 2 (Nrf2) molecule that sustains cellular redox homeostasis [6].

Diabetic cardiomyopathy is influenced by vascular endothelial growth factor (VEGF) that is responsible for blood vessels' formation to counteract cellular degeneration [7]. Concomitantly, downregulation of VEGF occurs with lowering of endothelial cells' apoptosis [8]. Treatment with erythropoietin (EPO) during cardiac ischemia decreases the chance of myocardial apoptosis [9]. EPO enhanced heart efficiency by incitement of endothelial ancestor cell-interceded endothelial turnover and VEGF upregulation [10].

*Nigella sativa* seeds (black cumin) have various beneficial pharmacological properties [11]. Thymoquinone (TQ; 2-isopropyl-5-methyl-1,4-benzoquinone) is a potent antioxidant phytochemical constituent present in *N. sativa* seeds that acts mainly by scavenging ROS and prevents cellular damage due to different prooxidants [12]. Herein, we investigated the defensive role of TQ against cardiomyopathy due to diabetes induction in rats regarding the intracellular pathway by which TQ may relegate diabetic cardiomyopathy.

## 2. Materials and Methods

### 2.1. Chemicals

Streptozotocin (STZ, S0130), dimethyl sulfoxide (DMSO, D2650), ethylenediaminetetraacetic acid (EDS), thymoquinone (274666), glucose (D9434), 0.1 M citrate buffer, phosphate-buffered saline (PBS, P5493), and sodium chloride solution (0.9%, 07982) were obtained from Sigma-Aldrich (St. Louis, MO, USA). *β*-Actin and Nrf2 antibodies were purchased from Santa Cruz Biotechnology Inc. (Santa Cruz, CA, USA).

### 2.2. Animals

The morals advisory group of Kafrelsheikh University, Egypt, permitted this study (KVM021/2016; March 2016). Sixty male rats weighing 180–200 g each were raised in the Physiology Unit, Faculty of Veterinary Medicine, Kafrelsheikh University, Egypt. Rats were maintained and fed in the constant conditions recommended in our previous work [13].

### 2.3. Experimental Design

Animals were assigned into four different groups (control, diabetic, diabetic + TQ, and TQ; 15 each), and every group was allocated into three repeats (5 each). Control and TQ groups were injected intraperitoneally (IP) once with 0.5 ml citrate buffer (pH 4.5) per rat. Diabetes was induced and monitored in diabetic and diabetic + TQ groups according to Atta et al. [13]. At the end of 12 weeks, the rats were anesthetized with intravenous infusion of sodium pentobarbital (30 mg/kg) to prevent suffering for accurate sampling.

### 2.4. Sampling

After 12 weeks, whole blood, serum, and heart samples for Western blot and RT-PCR were taken according to Atta et al. [13].

### 2.5. Biochemical Analysis

Kits from Merck (India Ltd.) were used for determination of plasma total cholesterol, triacylglycerol (TAG), and high-density lipoprotein-cholesterol (HDL-C). Low-density lipoprotein-cholesterol (LDL-C) was calculated by the method of Friedewald et al. [14]. Commercial ELISA diagnostic kits from BioCheck (Foster City, CA, USA) were used for determination of plasma troponin I (BC-1105) and creatine kinase-MB (CK-MB, BC-1121).

Heart inflammatory cytokines were assayed with ELISA kits for E-selectin (MBS762069, MyBioSource, San Diego, CA, USA), high sensitive C reactive protein (CRP) (MBS268328, MyBioSource), and interleukin-6 (IL-6) (MBS726707, MyBioSource). Malondialdehyde (MDA) concentrations in plasma and tissue homogenates were assessed using a thiobarbituric acid method [15]. Total superoxide dismutase (T.SOD) was measured in the plasma and cardiac tissue supernatant using nitro blue tetrazolium following the method of Nishikimi et al. [16]. Nitric oxide (NO) quantities in tissue supernatant were quantified according to the method of Miranda et al. [17].

Protein concentrations of cardiac homogenates were assessed with the Bradford assay (5000002, Bio-Rad Laboratories, Watford, UK) for calibration of biochemical assessment [18].

### 2.6. Assessment of Gene Expression

Total RNA contents were extracted from heart tissue samples in 1 ml QIAzol (79306, QIAGEN Inc., Valencia, CA, USA) with chloroform. The RNA pellets were rinsed with 70% ethanol, dried, and suspended in diethylpyrocarbonate (DEPC, 129112, QIAGEN Inc.). RNA amount and purity were assessed using a spectrophotometer at 260 nm. The ratio of the 260/280 optical density of all RNA tested was 1.7–1.9. RNA in samples was transcripted to the corresponding cDNA with RevertAid Premium reverse transcriptase (EP0733, Thermo Fisher Scientific, Deutschland, Germany). EPO, VEGF, and inducible nitric oxide synthase (iNOS) gene expressions' concentration were examined with RT-PCR using a Bio-Rad MJ Mini Opticon Real-Time PCR System. The primer sequences for EPO, VEGF, iNOS, and GAPDH (housekeeping) genes are listed in Table 1. Data are presented relative to control values using three separate experiments.

### 2.7. Western Blotting

Heart tissue was homogenized in ice-cold lysis buffer and then centrifuged at 14,000 ×g for 20 min at 4°C. Samples' protein amount was evaluated following Bradford [18]. Samples of equivalent protein amounts were subjected to electrophoresis using SDS/PAGE and transferred to PVDF membrane (88518, Thermo Fisher Scientific) after 1 h, and then blocked using 5% nonfat dried milk in Tris-Tween. The membrane was kept with a polyclonal rabbit anti-Nrf2 antibody (1 : 200, Santa Cruz Biotechnology) and anti-*β*-actin (Santa Cruz Biotechnology) as internal control diluted 1 : 1000 in Tris-Tween buffer. The membranes were treated with the secondary antibodies (1 : 3000) (611-1302, Rockland Immunochemical, Boyertown, PA, USA) incubated with membranes for 1 h at room temperature and rinsed. Protein bands were densitometrically assessed by means of Image J software version 1.48 (National Institutes of Health, Bethesda, MD, USA). Band density was normalized to the equivalent density of *β*-actin.

### 2.8. Histological Study

Heart samples were fixed in 10% neutral buffer formaldehyde (F8775, Sigma-Aldrich) solution for a minimum of one day. Fixed tissues were handled via paraffin embedding method and dehydrated via ascending sorts of ethanol (32205, Sigma-Aldrich), clearing in xylene, and immersed in paraffin (327204, Sigma-Aldrich), then implanted in paraffin wax at 60°C. Five *μ*m thick sections were dyed with hematoxylin and eosin [21]. Vacuolation percentages in the photomicrographs of all groups were quantified with Image J software.

### 2.9. Statistical Assessment

Variance analysis (one-way ANOVA) and results were subjected to Bonferroni's multiple comparisons post hoc test using GraphPad Prism 5 (GraphPad Software, San Diego, CA, USA) with *p* < 0.05 considered statistically significant. Results are shown as means ± standard error.

## 3. Results

### 3.1. Thymoquinone and Plasma Biochemical Factors Related to Cardiac Activity

**Figure 1:**
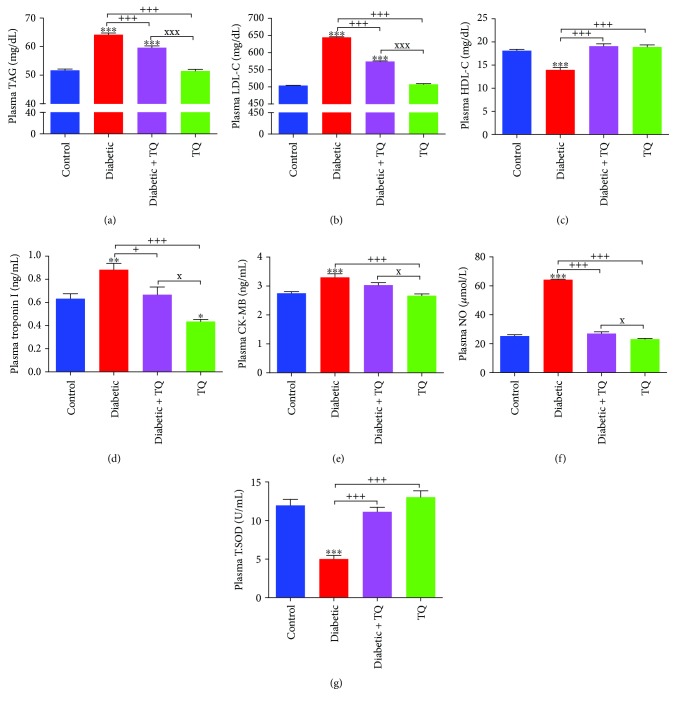
Plasma levels of TAG (a), LDL-C (b), HDL-C (c), troponin I (d), CK-MB (e), NO (f), and T.SOD (g). ^∗^*p* < 0.05, ^∗∗^*p* < 0.01 and ^∗∗∗^*p* < 0.001 vs. control. ^+^*p* < 0.05 and ^+++^*p* < 0.001 vs. diabetic. ^x^*p* < 0.05 and ^xxx^*p* < 0.001 vs. TQ. TQ: thymoquinone; TAG: triacylglycerol; LDL-C: low-density lipoprotein cholesterol; HDL-C: high-density lipoprotein cholesterol; CK-MB: creatine kinase-MB; NO: nitric oxide; T.SOD: total superoxide dismutase.

Plasma TAG, LDL-C, troponin I, and creatine kinase were significantly elevated in the diabetic rats although they returned to close to control group levels in the TQ group (Figure 1), while the diabetic group showed significant decreases in HDL-C compared to other groups as presented in Figure 1(c). Plasma NO was significantly elevated in the diabetic rats and was reduced in diabetic + TQ rats (Figure 1(f)). Plasma T.SOD activities were significantly reduced in the diabetic rats matched with control and TQ groups, which might be owing to damage due to diabetes (Figure 1(g)).

### 3.2. Thymoquinone and the Proinflammatory Markers of Cardiac Tissues

**Figure 2:**
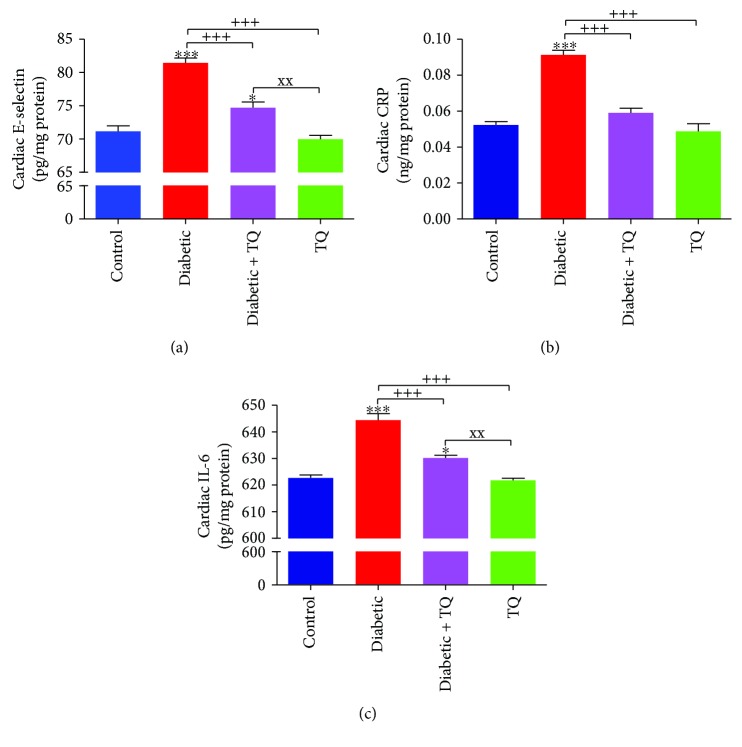
Cardiac levels of E-selectin (a), CRP (b), and IL-6 (c). ^∗^*p* < 0.05 and ^∗∗∗^*p* < 0.001 vs. control. ^+++^*p* < 0.001 vs. diabetic. ^xx^*p* < 0.01 vs. TQ. TQ: thymoquinone; CRP: C reactive protein; IL-6: interleukin-6.

The protecting influence of TQ on the inflammatory cytokines was evaluated. As presented in Figure 2, E-selectin, CRP, and IL-6 levels were significantly raised (*p* < 0.01) in the diabetic group vs. control and other treated rats.

### 3.3. Thymoquinone and Cardiac Tissue Antioxidant Status

**Figure 3:**
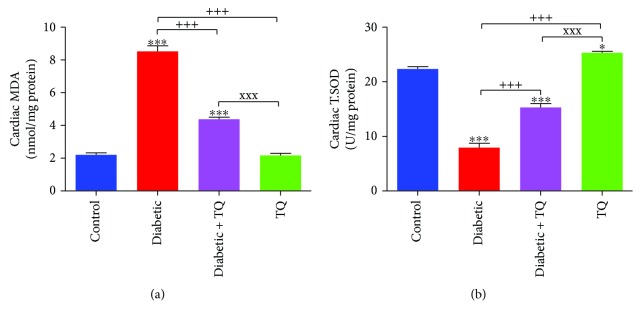
Cardiac MDA levels (a) and T.SOD activities (b). ^∗^*p* < 0.05 and ^∗∗∗^*p* < 0.001 vs. control. ^+++^*p* < 0.001 vs. diabetic. ^xxx^*p* < 0.001 vs. TQ. TQ: thymoquinone; MDA: malondialdehyde; T.SOD: total superoxide dismutase.

Diabetic rats displayed a significant increase (*p* < 0.001) in lipid peroxidation levels as monitored with levels of MDA, as well as a significant decrease (*p* < 0.001) in the activities of T.SOD as compared to control. Diabetic + TQ rats had significantly decreased levels of MDA (*p* < 0.001) and expressed a significant increase in T.SOD levels (*p* < 0.01). Conversely, the diabetic + TQ group showed significantly decreased (*p* < 0.001) T.SOD activity and significantly increased (*p* < 0.001) MDA level, significantly increased in comparison to control, as shown in Figure 3.

### 3.4. Thymoquinone and Cardiac EPO, VEGF, and iNOS mRNA Levels and the Nrf2 Protein Level

**Figure 4:**
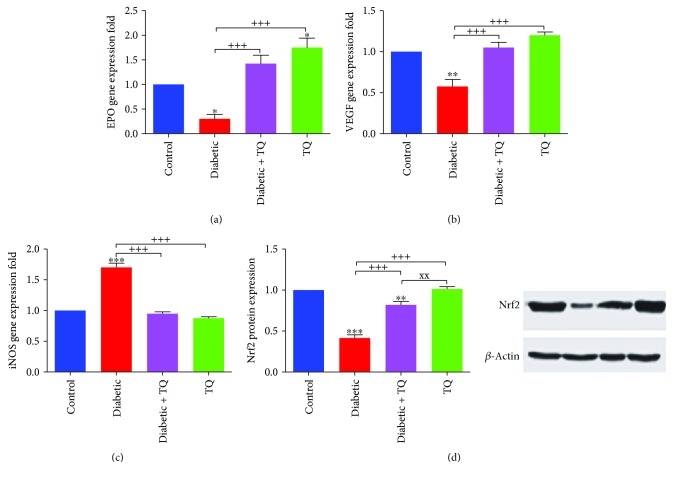
Gene expression folds of cardiac EPO (a), VEGF (b), and iNOS (c) genes and Western blot of Nrf2 (d). ^∗^*p* < 0.05, ^∗∗^*p* < 0.01, and ^∗∗∗^*p* < 0.001 vs. control. ^+++^*p* < 0.001 vs. diabetic. ^xx^*p* < 0.01 vs. TQ. VEGF: vascular endothelial growth factor; EPO: erythropoietin; iNOS: inducible nitric oxide synthase; Nrf2: nuclear factor erythroid 2-related factor 2.

The mRNA expression of EPO significantly decreased (*p* < 0.05) in the diabetic group while the EPO mRNA expression increased significantly (*p* < 0.05) in the diabetic + TQ group, even more than control (Figure 4(a)). The VEGF gene significantly decreased (*p* < 0.01) in the diabetic group, whereas in the diabetic + TQ rats, the VEGF mRNA expression was significantly increased (*p* < 0.001) in comparison to the diabetic group (Figure 4(b)). Cardiac iNOS mRNA was significantly elevated (*p* < 0.001) in the diabetic group vs. control, while in the TQ group, the level of iNOS expression was similar to control (Figure 4(c)). The protein level of Nrf2 assessed with Western blot is displayed in Figure 4(d). The diabetic group showed significant downregulation (*p* < 0.001) of Nrf2 protein expression in comparison to control and other TQ-treated groups. The diabetic + TQ group displayed upregulation of Nrf2 protein expression.

### 3.5. Histopathology

**Figure 5:**
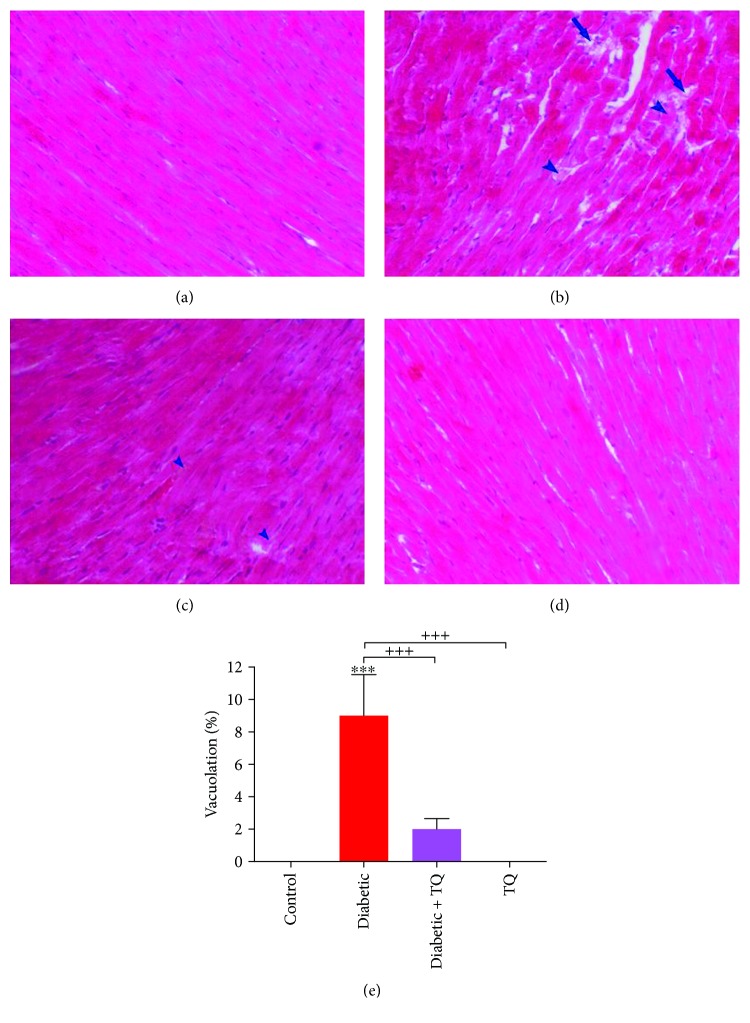
Photomicrograph of the rat myocardium showed normal myocardial fiber architecture in the negative control group (a) and TQ group (d). Photomicrograph of diabetic (b) and diabetic + TQ groups (c) showed myocardial degeneration (arrowhead) and vacuolation (arrow). H&E, ×200. (e) Vacuolation percentages. ^∗∗∗^*p* < 0.001 vs. control. ^+++^*p* < 0.001 vs. diabetic.

The histopathological assay of cardiac tissue from control and TQ groups showed regular morphological appearances, normal myocardial fiber structure, and architecture with no evidence of degeneration and vacuolation (Figure 5(a) and 5(d)). The myocardial sections of the diabetic group revealed marked myolysis and degeneration in addition to the vacuolation of myocardial fibers (Figure 5(b)). The myocardial sections of the diabetic + TQ group showed small areas of slight degeneration and vacuolation (Figure 5(c)). Compared to control, the vacuolation percentages in diabetic, diabetic + TQ, and TQ groups were 9.00 ± 2.54, 2.00 ± 0.65, and 0%, respectively (Figure 5(e)).

## 4. Discussion

Diabetes mellitus is a disorder accompanied by an increased glucose level and hyperlipidemia along with problems in insulin and erythrocytic hemoglobin glycosylation [22]. In this study, we have similar data of body weights, serum insulin levels, and HbA1c (%) as presented in our previous study [13], whereas the body weights and insulin concentrations in the diabetic group were significantly reduced compared to the control and TQ-treated rats. TQ caused significant improvement in the body weights in addition to the insulin pattern in the diabetic + TQ group as opposed to the diabetic group. The diabetic group had a significant increase in the HbA1c (%) compared to control and other rat groups. Additionally, there were no marked changes among the TQ and control groups.

Diabetic rats had a significant elevation in plasma TAG and LDL-C levels, while HDL-C levels were significantly reduced when compared to control rats. These findings are consistent with results of Ighodaro et al. [23] and Zhang et al. [24] in which the authors reported significant elevations in total cholesterol, TAG, HDL-C, and LDL-C of STZ-treated rats, and with our previous results that the plasma concentration of insulin was significantly reduced in diabetic rats, which led to hyperglycemia with a high percentage of HbA1C [13]. Alternatively, TQ significantly improved the plasma levels of TAG, LDL-C, and HDL-C in the diabetic + TQ group in relation to the diabetic group. Prabhakar et al. [25] reported the antihyperlipidemic consequence of TQ in contradiction to a high-fructose diet-induced metabolic disorder in rats. Also, TQ attenuated the significant increase in TAG and total cholesterol of cyclophosphamide-induced cardiomyopathies in rats [26]. We previously reported that TQ supplementation to diabetic rats reverted the plasma level of insulin and erythrocytic HbA1C to near their normal levels, indicating the importance of TQ in the regeneration of *β*-cells injured by STZ [13]. Concomitantly, TQ modulates hyperglycemia and decreases the rate of hemoglobin glycation.

Troponin I and CK-MB are plasma cardiac biomarkers that aid in the laboratory diagnosis of heart attack. STZ induced a significant increase in plasma troponin I and CK-MB, while TQ counteracted the oxidative injuries in cardiac muscles due to STZ. Giribabu et al. [27] stated that STZ-nicotinamide-induced cardiac injuries were accompanied by significant increases in troponin I and CK-MB in diabetic rats. On the contrary, TQ ameliorated the cardiac injuries that occurred in diabetic rats through hindering inflammatory progression and enhancing antioxidant status [28]. This experiment revealed that TQ potentiated the antioxidant status of plasma along with cardiac muscles of STZ-treated rats via significant decreases in plasma NO and cardiac MDA, while significantly increasing the plasma and cardiac T.SOD. Similarly, TQ significantly decreased MDA levels and increased T.SOD activities in *β*-cells of the STZ-induced diabetic group [29]. In addition, TQ significantly defeated oxidative damages induced by STZ in rats via significant decreases in testicular NO and MDA levels. Likewise, TQ increased testicular reduced glutathione levels and T.SOD activities, alleviating testicular injuries of diabetic rats [13].

Diabetes accompanied by inflammation and heart disorder is correlated with increased inflammatory biomarkers and cytokines [30], and therefore, plasma E-selectin, CRP, and IL-6 can be augmented in diabetic rats. Nawale et al. [28] stated that alloxan-induced diabetic rats had a significant level of CRP. Also, CRP, IL-6, E-selectin, and TNF-*α* were significantly raised in diabetic rats in response to oxidative damage [5, 31, 32]. On the contrary, TQ lowered the plasma levels of E-selectin, CRP, and IL-6. This result agreed with Karaca et al. [33], who noticed a significant reduction in interleukin-1 beta (IL-1*β*), IL-6, tumor necrosis factor-alpha (TNF-*α*), and monocyte chemoattractant protein-1 (MCP-1) in rats subjected to experimental induction of esophagitis. Also, it was found that TQ has a role against inflammatory progression in the hippocampal tissues due to lipopolysaccharide-induced inflammation in rats [34].

Here, the results revealed significant decreases of EPO and VEGF expressions in diabetic rats, while iNOS gene expression fold was significantly increased due to oxidative injuries of cardiac muscles. In the same context, diabetic rats had decreased renal mRNA expression of EPO as a result of oxidative stress due to diabetes [35]. Also, the VEGF gene was downregulated in cardiac tissue of diabetic rats, leading to impairment of angiogenesis [36]. Furthermore, iNOS expression was increased in STZ-diabetic group as shown by Nagareddy et al. [37]. Diabetic rats orally given TQ showed significant improvement of EPO and VEGF and reduction in iNOS genes that led to enhancement of heart angiogenesis and antioxidant status [13, 38].

**Figure 6:**
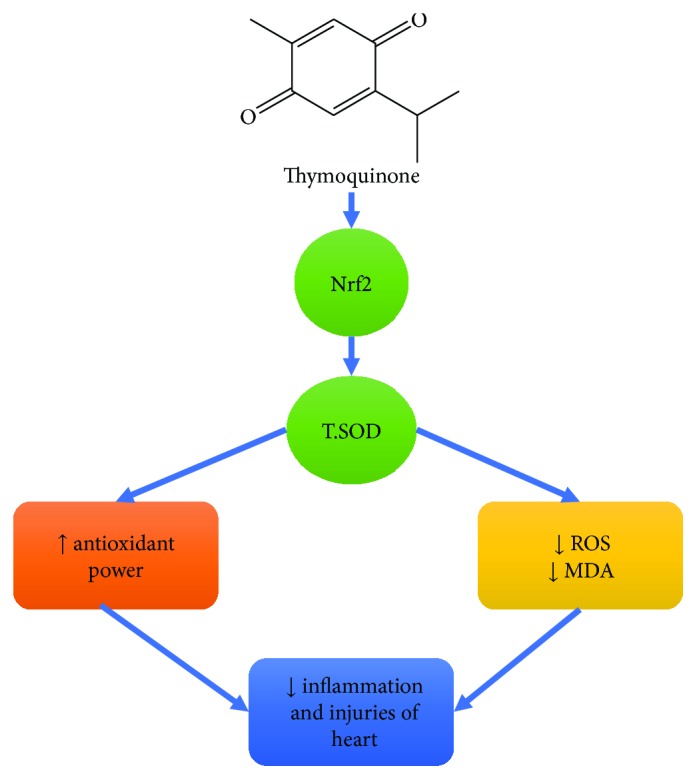
Scheme summarizing pathways involved in TQ attenuation of cardiomyopathy.

A major function of Nrf2 is defeating the oxidant stress that enhances the expression of cellular antioxidant molecules that guard against oxidative injury [39]. Nrf2 defeats ROS through induction of SOD and glutathione peroxidase (GPx) along with regeneration of oxidized glutathione (GSSG) [40]. Also, Nrf2 direct substrate and effector of protein kinase R- (PKR-) like endoplasmic reticulum kinase (PERK) mediated cell survival through amelioration of the endoplasmic reticulum and unfolded protein response (UPR) oxidative stresses [41]. Therefore, Nrf2 counteracts the cellular damages due to numerous injuries. The current study showed a considerable decrease in the Nrf2 protein level in diabetic rats relative to normal, control rats. High glucose-induced apoptosis in cardiomyocytes leads to depletion of Nrf2 and antioxidant status of STZ-diabetic rats [42]. Liu et al. [43] found that mulberry granules, a traditional Chinese medicine prescription, protect against STZ-induced cardiomyopathy by suppressing the oxidative stress through Nrf2. The current study displayed the defensive role of TQ counter to the oxidative stress caused by STZ through induction of Nrf2 and antioxidant enzymes (Figure 6). In a like manner, it was shown that the antioxidant potential of TQ through upregulation of Nrf2 consequently augmented the cellular antioxidant status and defeated the cellular oxidative injury [44]. Also, TQ has a crucial shielding role against DNA oxidative damage [45]. The upregulation of Nrf2 due to TQ is considered as a core of the TQ protective effect against cardiomyopathy in diabetic rats. Additionally, Liu et al. [46] suggested that the protective effect of the TQ on cardiovascular function might be due to downregulation of cyclooxygenase-2 levels and the increased phosphorylated-protein kinase B (p-Akt) expression levels in diabetic rats. These findings are mutually supportive to the cardioprotective effect of TQ against diabetic cardiomyopathy demonstrated in our study.

STZ induces oxidative injuries in cardiac muscles through increased production of ROS that leads to myolysis and degeneration in cardiac muscles and finally to failure. Wu et al. [47] reported a significant lessening in antioxidant enzymes with an increased concentration of MDA, leading to heart disease. On the contrary, TQ ameliorates the histopathological changes induced in rat cardiac muscles due to diabetes oxidative injuries and inflammation. Similarly, TQ protects the cardiac cells against injurious effect induced by different toxicants [48].

## 5. Conclusions

Diabetes exerts oxidative stress on the cardiac tissues by increasing the oxidative damage, mostly through elevated plasma NO and upregulation of cardiac tissue iNOS mRNA expression. Diabetes decreases the antioxidant ability of heart tissue by decreasing plasma and cardiac tissue T.SOD levels and downregulating cardiac tissue EPO and VEGF mRNA expressions, thereby increasing the major cardiac markers plasma troponin I and CK-MB. Oral administration of TQ protects the cardiac tissue from these oxidative stresses as manifested by normalization of the cardiac markers troponin I and CK-MB. This was achieved via improving the antioxidant status of cardiac muscle and upregulating VEGF and EPO expression, in addition to normalizing the protein expression of Nrf2. Briefly, TQ ameliorates the cardiac injuries in diabetic rats through upregulation of Nrf2 that alleviates oxidative stress and induces cell survival.

## Figures and Tables

**Table 1:** Primers for gene expression by RT-PCR.

Gene	Direction	Primer sequence	References
GAPDH	Sense	CAAGGTCA TCCATGACAACTTTG	[19]
	Antisense	GTCCACCACCCTG TTGCTGTAG	
EPO	Sense	TACGTAGCCTCACTTCACTGCTT	[19]
	Antisense	GCAGAAAGTATCCGCTGTGAGTGTTC	
iNOS	Sense	TCTGTGCCTTTGCTCATGAC	[20]
	Antisense	CATGGTGAACACGTTCTTGG	
VEGF	Sense	TATGTTT GACTGCTGTGGACTTGA	[19]
	Antisense	AGGGATGGG TTTGTCGTGT	

## Data Availability

The data used to support the findings of this study are included within the article.

## Abbreviations

ACP: Acid phosphatase
ALP: Alkaline phosphatase
EPO: Erythropoietin
HDL-C: High-density lipoprotein cholesterol
iNOS: Inducible nitric oxide synthase
LDL-C: Low-density lipoprotein cholesterol
MDA: Malondialdehyde
NO: Nitric oxide
ROS: Reactive oxygen species
STZ: Streptozotocin
T.SOD: Total superoxide dismutase
TAG: Triacylglycerol
TQ: Thymoquinone
VEGF: Vascular endothelial growth factor.
